# Exploration of Analysis Methods for Diagnostic Imaging Tests: Problems with ROC AUC and Confidence Scores in CT Colonography

**DOI:** 10.1371/journal.pone.0107633

**Published:** 2014-10-29

**Authors:** Susan Mallett, Steve Halligan, Gary S. Collins, Doug G. Altman

**Affiliations:** 1 Department of Primary Care Health Sciences, University of Oxford, Oxford, United Kingdom; 2 Centre for Medical Imaging, University College London, London, United Kingdom; 3 Centre for Statistics in Medicine, University of Oxford, Oxford, United Kingdom; Queensland Institute of Medical Research, Australia

## Abstract

**Background:**

Different methods of evaluating diagnostic performance when comparing diagnostic tests may lead to different results. We compared two such approaches, sensitivity and specificity with area under the Receiver Operating Characteristic Curve (ROC AUC) for the evaluation of CT colonography for the detection of polyps, either with or without computer assisted detection.

**Methods:**

In a multireader multicase study of 10 readers and 107 cases we compared sensitivity and specificity, using radiological reporting of the presence or absence of polyps, to ROC AUC calculated from confidence scores concerning the presence of polyps. Both methods were assessed against a reference standard. Here we focus on five readers, selected to illustrate issues in design and analysis. We compared diagnostic measures within readers, showing that differences in results are due to statistical methods.

**Results:**

Reader performance varied widely depending on whether sensitivity and specificity or ROC AUC was used. There were problems using confidence scores; in assigning scores to all cases; in use of zero scores when no polyps were identified; the bimodal non-normal distribution of scores; fitting ROC curves due to extrapolation beyond the study data; and the undue influence of a few false positive results. Variation due to use of different ROC methods exceeded differences between test results for ROC AUC.

**Conclusions:**

The confidence scores recorded in our study violated many assumptions of ROC AUC methods, rendering these methods inappropriate. The problems we identified will apply to other detection studies using confidence scores. We found sensitivity and specificity were a more reliable and clinically appropriate method to compare diagnostic tests.

## Introduction

Comparisons of diagnostic tests aim to inform healthcare providers and patients which tests are most accurate. The ideal test would give all patients a correct diagnosis, in a short time and with minimal inconvenience to the patient. Unfortunately no test is perfect, and in practice some patients with the target disease will be missed (false negative result), and some patients without disease will be diagnosed incorrectly with disease (false positive result).

### Measuring diagnostic performance

There are three main approaches for comparing diagnostic test accuracy that use different statistical measures. In a previous paper we have discussed these approaches with illustrative examples [1]. The first approach is to use paired measures at specific test thresholds, using either sensitivity and specificity, positive predictive value and negative predictive value (PPV and NPV), or positive likelihood ratio and negative likelihood ratio (LR+ and LR−). A second approach is to examine test performance across all diagnostic test thresholds, using summary measures such as ROC AUC or diagnostic odds ratio (DOR). A third approach gives an overall measure at a specific threshold (or series of thresholds), reported alongside the paired measures for example using a weighted comparison measure [2], [3] or net benefit [4], [5]; using a single measure can be to simplify comparisons of overall results compared to using paired measures that are likely to change in different directions, of which sensitivity and specificity are the best known examples.

### Multi-Reader Multi-Case designs

In radiology, multi-reader multi-case (MRMC) studies are often used to compare the diagnostic accuracy of alternative imaging approaches and this design is currently required by the United States Food and Drug Administration (FDA) for pre-market evaluation [6]. Key attributes of good study design are uncontroversial and include interpretation of medical images from the clinical population of interest by radiologists typical of those who would read the test in clinical practice, and unaware of the patient disease status or prevalence of abnormality. Studies often compare test interpretation by the same radiologists in the same patients, with the only difference being the diagnostic test. Multi-reader multi-case studies can either use a fully crossed design, where all readers interpret all patient images or split-plot designs [7]. Learning and order bias are reduced by presenting images and tests to each reader in random order. Interpretation of the same case is often separated by at least one month to reduce potential for recall bias.

### Clinical utility of CT colonography

Computed tomography (CT) colonography is a CT scanning technique used to identify colon polyps, the precursor of colon cancer. Diagnostic improvement occurs when correct detection of patients with polyps increases (false negative results are reduced), corresponding to an increase in sensitivity, without an unacceptable increase in false positive diagnoses, corresponding to a decrease in specificity. It is important to take disease prevalence into account when balancing changes in sensitivity and specificity. We have recently measured the relative value that patients and clinicians place on false positive results compared to false negative results using discrete choice experiments [8]. Both patients and medical professionals valued reducing false negative (increasing sensitivity) more desirable than reducing false positive results (reduction in specificity) for both colon polyps and colon cancer [8]. Similarly when in mammography screening women will exchange 500 false-positives for one additional cancer [9]. This is pertinent to ROC AUC, where the analysis automatically sets a weighting of the relative importance of diagnoses [1].

Sensitivity and specificity are usually direct measures calculated from diagnostic data reported by radiologists in normal clinical practice, namely the presence or absence of polyps. By contrast ROC AUC is a summary measure of performance across all potential diagnostic thresholds for positivity, rather than performance at any specific threshold. As such ROC AUC is classified as a surrogate endpoint [10].

### ROC AUC requires confidence scores

ROC AUC is derived from confidence scores which are scores usually assigned by radiologists to indicate their confidence in their diagnosis. Confidence scores may or may not form part of the normal clinical report. Confidence scores can be assigned either to individual lesions within a patient, or to an overall patient diagnosis.

In imaging studies there are two broad types of clinical scenario in which confidence scores can be assigned to enable calculation of ROC AUC. In “classification” studies, visualised lesions are classified according to morphological characteristics perceived by the radiologist; for example in mammography studies lesions are either benign or malignant and the strength of the radiologist's belief is captured using a confidence scale such as ‘benign’, ‘probably benign’, ‘equivocal’, ‘probably malignant’, or ‘definitely malignant’. If there is a lesion on every image presented, then the task is purely classification. In some studies the confidence score is adapted from a clinical measure used in clinical practice, such as the BI-RAD scale [11].

In “detection” or “presence versus absence” studies, readers are asked to record their confidence regarding the presence or absence of a lesion rather than its nature; often a scale such as 0 to 100 is used. These confidence scores are often recorded in clinical trials solely to calculate ROC AUC. It has been suggested that lesion size could act as a confidence score for “presence/absence studies” linked to normal clinical practice. However this approach is flawed as lesion size cannot be measured when there is no lesion.

Many studies are hybrids between these two scenarios. For example not all images may contain a lesion, and readers may be asked to classify lesions when present and use a different confidence score when not. Similarly “detection” studies may require readers to report confidence scores for abnormalities that they do not classify as lesions.

### Aim of research

In this paper we compare two statistical methods for measuring diagnostic performance, namely sensitivity and specificity versus ROC AUC. When used to compare two diagnostic tests these methods may estimate diagnostic performance differently. In this article we investigate why this can happen using data from a previously published clinical study [12] and examine which aspects of study design and characteristics of the data contributed to ROC AUC method assumptions being considered inappropriate.

We illustrate the issues using a study comparing CT colonography with and without Computer Assisted Detection (CAD) to identify colon polyps [12]. We compare the diagnostic measure area under the Receiver Operating Characteristic Curve (ROC AUC) to sensitivity and specificity. This work was motivated by an FDA strong presumption in favour of using ROC AUC to measure diagnostic accuracy for licensing of CAD in radiological imaging [6]. We identify and present the problems encountered when using ROC AUC to measure diagnostic performance.

## Methods

### Study design

Full methods for the study are described in the original study publication [12]. In brief, ten radiologists each read CT colonography images from the same 107 patients, reading images with and without CAD assistance to detect colon polyps. Each read was separated by two months to avoid potential recall bias, with both test and patient order randomised for each reader. The reference standard was a consensus of two from a panel of three experienced and independent radiologists who read each case combination with colonoscopy reports: 60 patients had polyps and 47 were normal.

Each reader identified polyps, noting their diameter and location. In addition they recorded whether they believed the patient case was normal (i.e. no polyps were seen) or abnormal (where polyps were reported). All statistical measures were calculated per patient since a positive CT colonography will mean subsequent colonoscopy (where the entire colon is examined and polyps removed). Sensitivity was the percentage of patients identified by radiologists as having a polyp, either through true positive or false positive polyp identification(s), from patients positive according to the reference standard. This definition of sensitivity reflects that patients are referred based on identification of polyps in the clinical referral pathway. Specificity is the percentage of patients where no polyps were reported by radiologists, of those classified as negative by the reference standard. Table 1 shows the steps used to calculate ROC AUC in this study. A confidence score between 1 and 100 was reported for each potential polyp identified, with readers instructed to use scores of 25 or above for polyps with high confidence and scores of 1 to 24 for abnormalities believed more likely to be something else. Where no confidence score was recorded by radiologists, a zero score was introduced during statistical analysis. Where more than one polyp was recorded per patient, the highest confidence score recorded with each patient was used for analysis. ROC AUC calculations used DBM MRMC v2.1 (http://www-radiology.uchicago.edu/krl/KRL_ROC/software_index6.htm) and Proproc v0.0 (http://metz-roc.uchicago.edu/MetzROC/software/software) [13]. DBM MRMC fits ROC curves based on parametric binormal methods [14]. PROPROC fits ROC curves based on a maximum-likelihood estimation using a proper binormal distribution [13]. In this paper, for illustrative purposes, we selected five of the ten readers that best demonstrate issues when comparing sensitivity and specificity versus ROC AUC.

**Table 1:** Steps in calculation of ROC AUC.

**Step A: Assigning confidence scores**
• Confidence scores were assigned by radiologists. Missing values assigned a value of zero by the data manager or statistician.
**Step B: Building the ROC curve from confidence scores and calculating ROC AUC**
• Distributions of the confidence scores were examined. Evaluation of potential limitations arising from non-normal distributions or extreme values.
• Real data points directly generated from confidence scores presented in ROC space
• ROC curves fitted using both parametric and nonparametric methods and examination of differences in resulting ROC AUCs. Evaluation of sensitivity of ROC curve to key values (such as values of confidence scores if few false positives), especially important as there are few false positive results.
• ROC AUC calculated using both parametric and nonparametric methods
**Step C: ROC AUC averaged across multiple readers and cases**
• Different models using fixed and random effects used to model data
• Random effects with 95% confidence intervals modelled by resampling (bootstrap [40]). Alternative methods can include jackknife [41]), permutation [42] or probabilistic method [43].

## Results

### Different diagnostic performance

**Figure 1:**
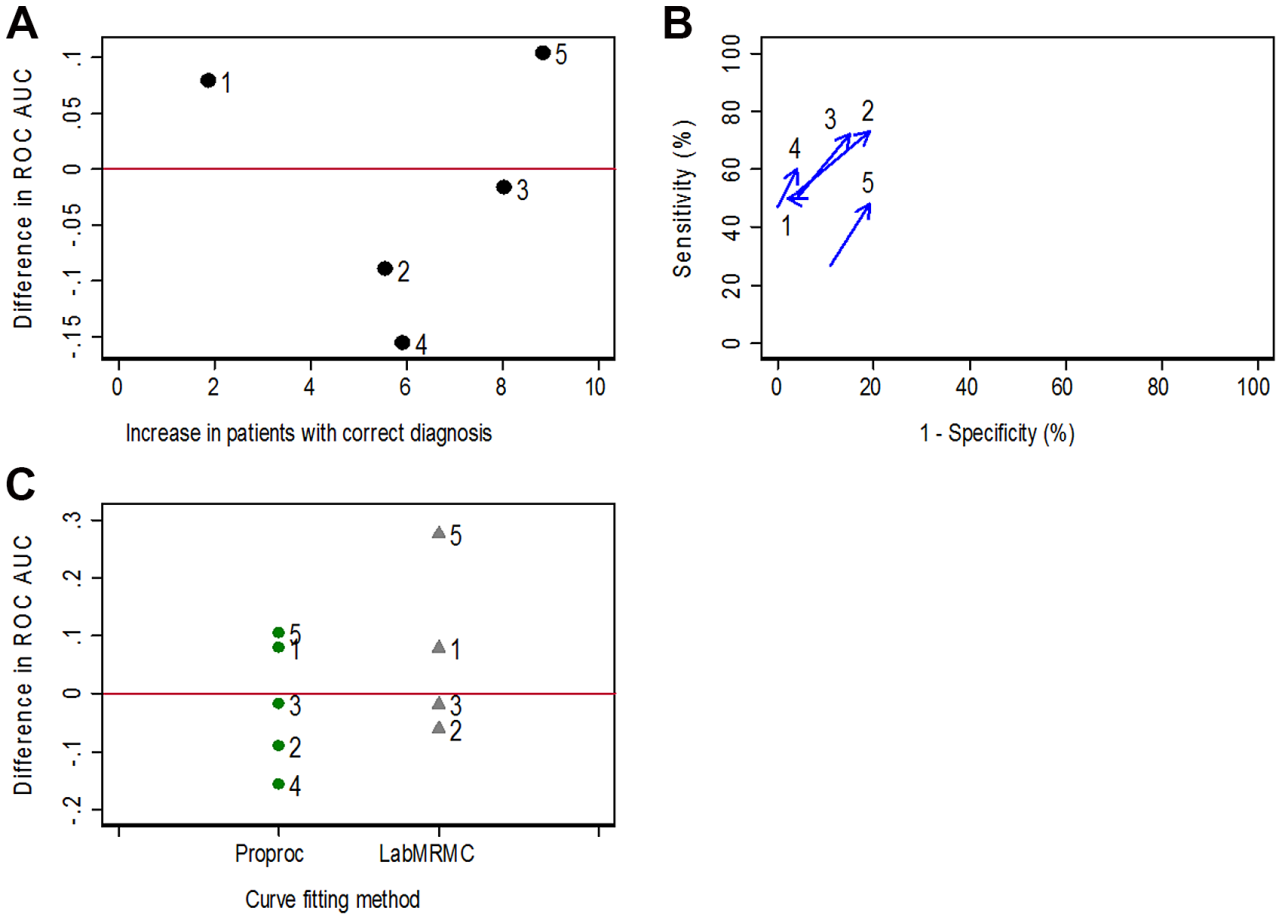
Difference in diagnostic performance of two tests showing readers from a multi-reader study. Change in diagnostic performance of CT colonography for the detection of polyps; difference with computer assisted detection (CAD) minus without CAD. Results from individual readers. A. Comparison of increase in the number of patients with a correct diagnosis with change in ROC AUC. The five readers selected for illustrative purposes as examples for the rest of the article are labelled from 1 to 5. B. Arrows indicate values of sensitivity and specificity for each reader, the arrow bases showing unassisted read values and the arrow head the CAD assisted read values for the same reader. C. Difference in ROC AUC using two methods for fitting ROC curves. ROC AUC could not be calculated for reader 4 using LabMRMC method.

We compared the diagnostic performance of two tests to detect colonic polyps, CT colonography either with or without CAD, using the difference in diagnostic accuracy measured by (i) the number of patients correctly diagnosed and (ii) ROC AUC. We expected diagnostic performance to increase when CAD was used. However, we observed no clear relationship between these two measures of diagnostic performance despite readers and cases being identical (Figure 1A).

We then investigated the relationship between the difference in sensitivity and specificity and the difference in ROC AUC (ΔROC AUC) for individual readers, focussing on five of the ten readers as illustrative examples (Figure 1B & 1C). Readers 2, 3 and 5 exhibited clear gains in sensitivity of 21, 22 and 21%, along with decreases in specificity of 15, 11 and 8% respectively. Reader 5 had the best performance followed by readers 3 and 2 respectively. Reader 4 also had a 13% increase in sensitivity with a smaller 4% decrease in specificity. Reader 1, by contrast, showed no increase in sensitivity but unusually had a 4% increase in specificity. Use of CAD improved clinical diagnosis in readers 2 to 5 but not in reader 1, based on the large increases in sensitivity when using CAD. As noted above, these are considered more important to both clinicians and patients than smaller reductions in specificity [8]. By contrast, the change in ROC AUC (Figure 1B) defines a positive benefit of CAD in readers 1 and 5 and a negative benefit in readers 2, 3 and 4. Perversely, reader 1 had one of the highest increases in ROC AUC (Figure 1A and 1B) since CAD had no influence on sensitivity, the most clinically important aspect, and also had little impact on specificity.

### Problems recording confidence scores that cause zero values

During our study readers encountered several problems when assigning the confidence scores needed to derive ROC AUC. A key problem was that radiologists only reported confidence scores for regions of the colon where they identified polyps, despite instructions to use confidence scores between 1 and 25 to report irregularities that were, on balance, likely not polyps. CT colonography of a normal colon identifies many potential abnormalities that are ultimately proven not to be polyps, often numerous, and it was impracticable to score all of these or to select a meaningful subset to score. Further, when an abnormality believed to be a polyp was encountered, it tended to be reported with high confidence. In order to include all patients in the study, the statistician or data manager assigned a value of zero when a confidence score was not assigned by a radiologist.

**Figure 2:**
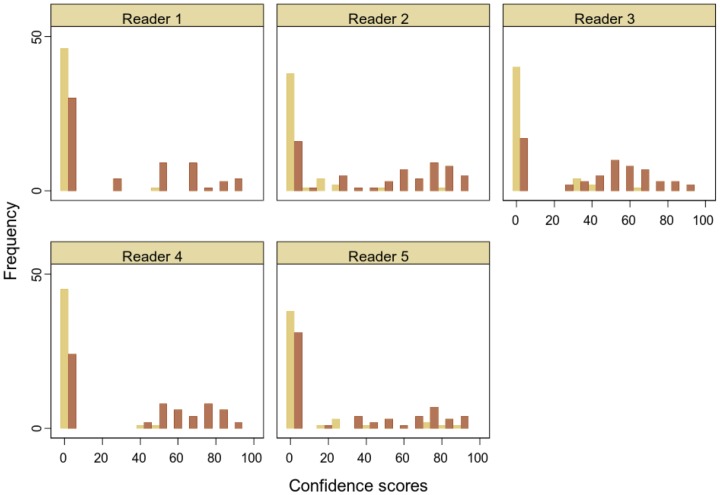
Distribution of confidence scores for patients with and without polyps. Each histogram shows the distribution of confidence score values using CAD CT colonography for an individual reader separately for patients with (brown) and without polyps (yellow) based on the reference standard. Five readers are shown in plots labelled 1 to 5.

Figure 2 shows the distribution of confidence scores for five readers. The most common score for every reader was zero. This zero-inflated “spike” then accompanies a second distribution of the confidence scores assigned for abnormalities believed to be polyps. This results in a bimodal distribution of confidence scores that cannot be not transformed to a normal distribution by simple data transformations used in standard open source software [15] developed for these analyses (Figure 2). Despite instructions in which distinct ranges of scores were linked to descriptions of confidence, each reader interprets the guidance differently and uses the scores in different ways.

### Examples of distributions of confidence scores from literature

**Figure 3:**
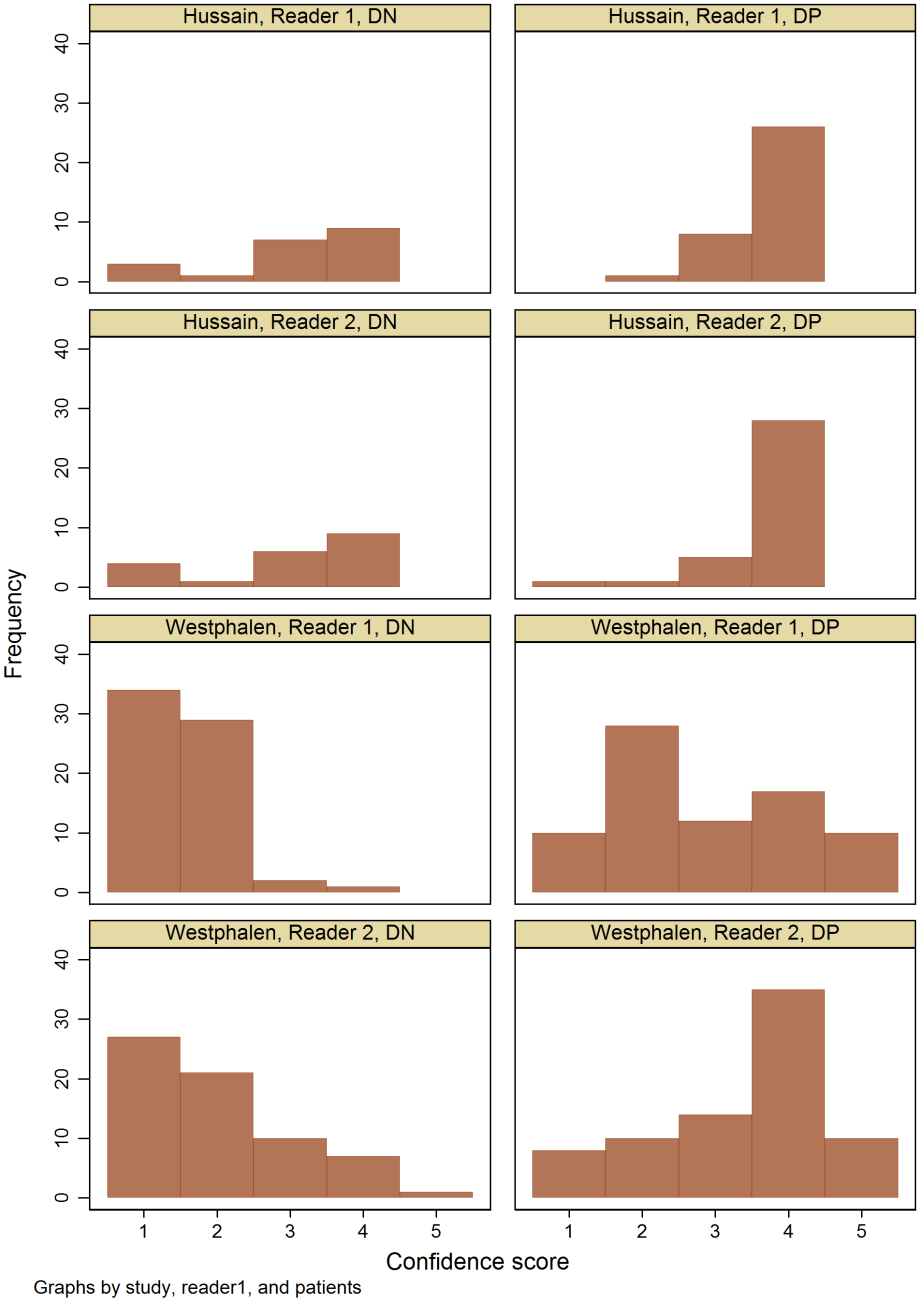
Distribution of published confidence scores. Published confidence scores assigned by individual readers (Reader 1 and Reader 2) reported for patients without disease (DN) and patients with disease (DP) from two studies. In Hussain et al. [17], classification of MR imaging of cirrohotic liver used categories: 1 definitely benign; 2 probably benign; 3 possibly malignant; 4 definitely malignant. In Westphalen et al. [18], classification of MR imaging of peripheral zone tissue from patients with prostate cancer used categories: 1 likely benign; 2 possibly benign; 3 equivocal or indeterminate; 4 possibly malignant; 5 likely malignant.

Very few published articles using MRMC ROC AUC report the distribution of confidence scores. We identified only two examples from the literature where individual reader scores were reported and another where the distribution of scores across the group of readers was shown (Figure 3 [16]–[18]). These examples show clearly that the distribution of confidence scores is not close to normality in either patient group, with or without monotonic transformation. However they generally have one peak (unimodal) rather than distributions with more than one peak, as observed in our study.

Authors of two other studies where confidence scores were used to rate the presence or absence of polyps in CT colonography, reported that scores were non-normal [19] [20]. Baker et al. stated this as a reason why MRMC ROC AUC analysis was not used [19]. Petrick et al noted that the lack of normally distributed scores led to empirical ROC analysis only via bespoke alteration to the software used, as standard parametric binormal ROC curve fitting could not be used [20].

### Position of data points on a ROC curve

**Figure 4:**
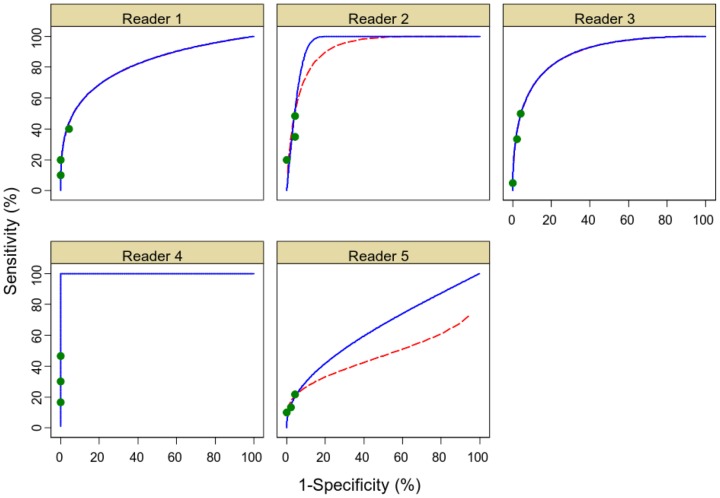
Different curve fitting methods. ROC plots each for an individual reader using CT colonography without CAD. Green dots indicate real data points underlying curve fitting. ROC curve are shown extrapolated from these data using DBM MRMC (red dotted line) and PROPROC software (blue solid line). Five readers are shown in plots labelled 1 to 5.

ROC curves are constructed by calculating sensitivity and specificity at all possible thresholds of confidence score to define a positive test result. Figure 4 shows the actual data points underlying ROC curves in our study. Fitting a ROC curve across all values of specificity requires extensive extrapolation beyond the study data with the result that the area underneath the extrapolated curve dominates ROC AUC rather than being driven by observed data.

### Fitting a ROC curve and calculating ROC AUC

Different methods can be used to fit ROC curves. These can generate different curves from the same study data and so produce different ROC AUCs. Figure 4 shows two curve fitting methods available in the Metz programs; the DBM MRMC method based on parametric binormal methods [14] (dotted line), and the PROPROC method based on ‘proper’ binormal distributions [13] (solid line). Non-parametric methods can also be used [21]. For readers 2 and 5 the extrapolated portion of the ROC curves differ greatly, demonstrating how the fitting method chosen can influence the calculated ROC AUC. Further, for reader 4 the DBM MRMC method was unable to fit a curve, as there were no false positive diagnoses. Table 2 shows ROC AUCs generated from these two methods and also using the Wilcoxon method, which is a non-parametric method that makes no assumptions regarding data distribution and which can be calculated without fitting any ROC curve. It should be noted that in many published clinical studies, the difference in ROC AUC between two tests being compared tends to be small, in the region of 0.07 [22], [23].

**Table 2:** ROC AUC using different methods and different ROC curves.

Reader	Wilcoxon ROC AUC (SE)	DBM MRMC ROC AUC (SE)	PROPROC ROC AUC (SE)
1	0.724 (0.048)	0.814 (0.123)	0.814 (0.120)
2	0.737 (0.048)	0.922 (0.073)	0.952 (0.034)
3	0.732 (0.048)	0.887 (0.105)	0.887 (0.105)
4	NC (NC)	NC (NC)	1 (NC)
5	0.589 (0.055)	0.469 (0.141)	0.641 (0.046)

ROC AUC and standard errors calculated for five readers using CT colonography without CAD.

**Figure 5:**
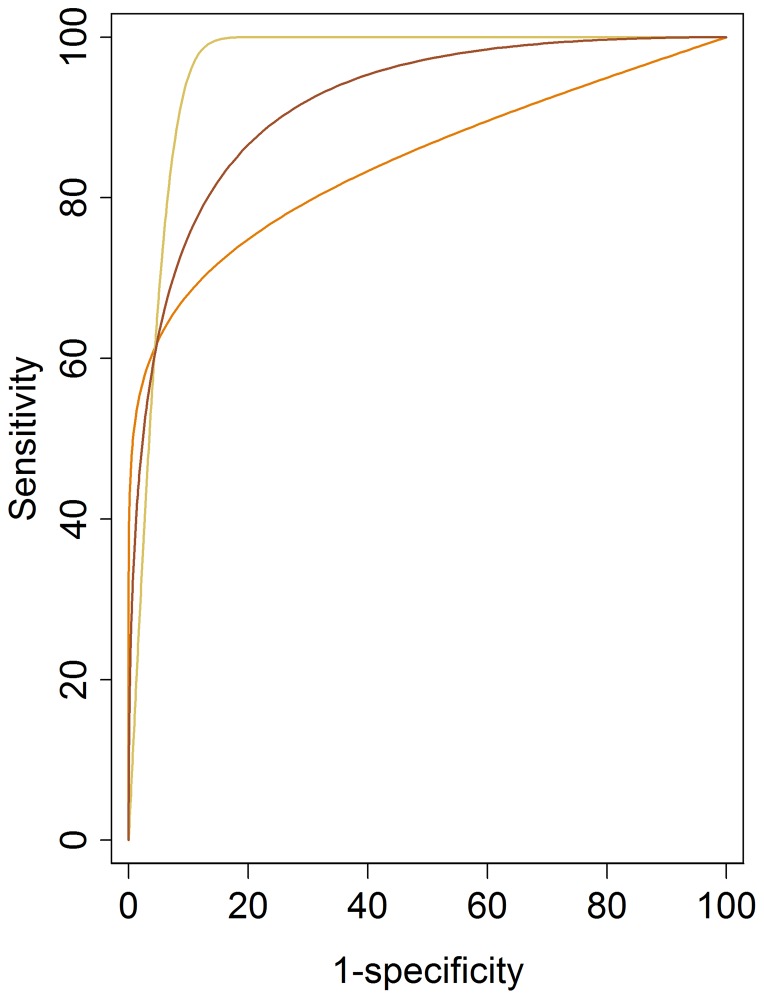
Impact of few false positives. ROC curve for reader 4 using CT colonography with CAD. The data from the original read (orange curve) includes two patients where false positive polyps have been indicated with confidence scores of 40 and 50. Perturbing these two patient scores to values of 70 (yellow curve) and 20 and 70 (brown curve) demonstrate changes in ROC curves. Sensitivity and specificity are expressed as percentages.

The Wilcoxon method is equivalent to the Wilcoxon statistic, an empirical method not requiring a ROC curve to be fitted. DBM and PROPROC methods sometimes give different ROC AUC because different ROC curves are fitted as seen in figure 5 for Readers 2 and 5. NC = could not be calculated with DBM MRMC software.

### Impact of few false positives on ROC AUC

A further undesirable characteristic of the surrogate endpoint ΔROC AUC in our study, was the large difference in ΔROC AUC precipitated by small differences in the confidence scores ascribed to false positives. Figure 5 shows the ROC curves of one reader (reader 4 in other figures) with two false positive detections with confidence scores of 40 and 50 respectively (reader 4 figure 2). This corresponds to the curve in orange and a ROC AUC of 0.84. Artificially increasing these two confidence scores to 70 increases the ROC AUC to 0.96 (yellow curve), whereas changing them to 20 and 70 respectively results in an ROC AUC of 0.92 (brown curve). Thus the shape of the ROC curve is heavily dependent on the relative ranking of confidence scores ascribed to two false positive patients versus true positive diagnoses. In our study the median number of patients with false positive scores per reader in unassisted reads was 2 of 107 patients, demonstrating how ROC AUC may be influenced heavily by a very small proportion of the observed data.

## Discussion

### Standard measures and ROC AUC do not correlate

We compared two diagnostic measures: ROC AUC, the percentage of patients with a correct diagnosis, and sensitivity and specificity, to quantify the impact of using CT colonography with and without CAD. Each measure was calculated for interpretation of the same patients by the same reader.

Diagnostic performance between imaging tests is often assessed by the difference in ROC AUC, which is the measure preferred by the FDA for regulatory licensing [6]. We favour using the difference in sensitivity and difference in specificity, as these can calculated using test measurements made in clinical practice, which are direct measures measured from real patients (i.e. without need for confidence scores). By contrast ROC AUC is a surrogate measure, as it does not assess test performance at a relevant clinical threshold, but instead assesses performance averaged over all possible thresholds.

In our study, the change in ROC AUC was not correlated with the change in the proportion of patients correctly diagnosed, for the same readers interpreting the same patients (Figure 1). Readers in whom a decrease in ROC AUC indicated decreased accuracy when using CAD actually exhibited increased accuracy when assessed by the difference in sensitivity and specificity (Figure 1B and 1C).

### Problems with confidence scores of zero

ROC AUC analysis requires confidence scores of diagnostic certainty to build the ROC curve. In our study we found that radiologists did not assign confidence scores in patients in whom they detected no abnormality. A zero score was therefore assigned by the study statistician in order to include all patients in the analysis (Figure 2). These scores then had an adverse effect on the analysis in several ways:

Firstly, some scores are default values of zero when no score was defined by radiologists in this study. Less obviously, the zero scores are a score based on whole patient diagnosis at an almost infinite number of locations in CT colonography where an abnormality might have been detected, whereas when an abnormality is detected the score is based on that specific region of interest within the image. Also, radiologists interpret and assign scores differently, despite receiving the same scoring instructions.

Secondly, there are often two types of true negative scores. In our study true negatives could result either because the colon wall was perceived to be normal or when an abnormality was perceived but was correctly identified (and scored) as not being a polyp. In a mammographic comparison of digital and film techniques, two types of true negative findings are also described; the first where no finding was identified and the second where a finding was identified but was thought to be benign rather than malignant [11]. Study designs where every image includes a lesion may have only one type of true negative (e.g. a lesion is seen but is believed to be benign rather than malignant), but most other studies are likely to have more than one type of true negative if they include patients with no abnormality.

A third issue is that in studies comparing two tests, there is an expectation that a better test will increase confidence scores. However for true negative results due to “normal” patients there is no room for improvement since a score of zero cannot be decreased further. Where there are a large number of such true negatives, there is little ability to demonstrate any diagnostic improvement.

A fourth and important issue is that zero scores mean that the basic requirement for a ROC curve to summarise all data in a valid fashion is broken, namely that the data is normally distributed or can be transformed to a normal distribution by a monotonic function [24]. In our study, the data from all patients where a zero score was assigned only contribute to the point at the origin of the ROC curve at the top right hand corner of the plot and so do not contribute to the shape of the curve. The ROC AUC then becomes a summary of a subset of the study data and, as in our study, is likely not to include data from a large proportion of patients in whom no abnormality was detected. These patients include both those with a true negative result and those with a false negative result. In our study, due to high specificity indicating good test performance, 80% to 100% of TN patients were not included in the ROC curve. In addition due to a lower sensitivity between 28% to 77%, FN patients were also excluded. Overall, only 15% to 47% of the 107 patients in the study actually contributed to the ROC curve and, therefore, the ROC AUC. Harrington pertinently highlights this as a key issue, noting that ROC AUC is silent on false-negative and true-negative diagnoses despite their substantial clinical importance [25]. The distribution of confidence scores in our study was bimodal and resembles the distribution of rainfall data, where there are definite zero values (days on which there is no rain) together with continuous scores (reflecting the amount of rain on those days when it does rain). Problems modelling rainfall data are well-recognised, due to the lack of continuity between the zero scores (binary data) and those that are continuous. Thus rainfall models are often split; one model describes the probability that a day is wet or dry (binary data), and a second describes the amount of rain when the weather is wet (continuous data) [26].

There are significant issues with modelling data that is neither solely discrete nor continuous. Accordingly, we feel it is inappropriate to model such data in the simple binormal format to calculate a ROC curve.

### Other problems with confidence score distribution

The distribution of confidence scores can also cause problems even where there are no problems with zero scores. ROC AUC was introduced to medical diagnosis [27] based on its ability to distinguish between two diagnostic alternatives (with and without target condition or disease) and assuming the distributions of confidence scores are normally distributed for patients both with and without disease. However most radiological tests are clinically useful because they facilitate identification of patients with disease or ruling out patients who don't have disease, and so the distributions of confidence scores for either or both sets of patients are usually not normally distributed. Disappointingly, most papers that cite the MRMC using DBM MRMC or Metz software do not report the distribution of confidence scores in their data. Figure 3 shows two examples of distributions of confidence scores from our study, which demonstrate the characteristic of extreme scores in patients either with or without disease. Hanley has shown that for classification studies with a small number of rating categories, ROC curves can be reasonable under a mixture of normal distributions even for data with highly non normal distributions [28]. However others have raised concerns for ROC AUC analysis for tests intended to identify well defined abnormalities [29] (i.e. tests where there are few false positive results such as diagnosis of ankle fractures using single view radiographs [30]). With well defined abnormalities, there are two issues: firstly the distribution of confidence scores is highly non-normal and cannot be transformed to a normal distribution, and secondly there may be few false positive results that then lead to curve fitting problems (see figure 5). Both of these components lead to a situation where confidence scores for patients with and without disease do not have sufficient overlap to fit a reliable ROC model. Where there is little overlap, then confidence scores essentially represent binary decisions, possibly with an equivocal category. Reviewing several of his studies for detection of well defined abnormalities, Gur found that with a few exceptions, more than 90% of interpretations had scores at the extremes of the range (i.e. greater than 89% or less than 11% on the confidence scale) [29]. Gur suggested this might be a particular characteristic of detection studies, as opposed to characterisation studies. Dorfman et al have also identified issues when using ROC AUC to analyse tests that dichotomise into clearly positive and negative results, and so generate few false positives [30].

One suggestion is that when confidence scores are not normally distributed, readers are re-trained to spread their confidence scores across the full range of the available scale [31]. We share the concerns expressed by Gur that this process intended to achieve a better spread of data specifically for ROC analysis raises doubts about subsequent generalisability of findings to the clinical environment [29]. We also note that the ranking methods used to analyse confidence scores in DBM MRMC software acts to condense and reduce the difference between scores in cases where there is good separation of scores due to well-defined abnormalities. These ranking methods will therefore undervalue the discrimination of better tests [32] whereas tests with poorer discrimination between confidence scores will be overvalued. Unfortunately, the better the test, the worse ROC methodology performs as an analysis tool due to confidence scores being concentrated at extreme values or violating distributional assumptions. Perversely, the worse the imaging test, the better these statistical methods make it appear.

Although studies using typical characterisation categories such as ‘normal’, ‘probably normal’, ‘equivocal’, ‘probably abnormal’, or ‘definitely abnormal’ may avoid problems with normality assumptions [28], other key issues arise since these categories do not conform to an evenly spaced ordinal score giving an ROC AUC value which is harder to interpret [25]. Some scoring systems such as BI-RADS (Breast Imaging-Reporting and Data System) have been analysed using ROC AUC but are not ordinal. A BI-RADS rating of 2, defined as benign abnormality, does not imply a greater suspicion of cancer than a BI-RADS rating of 1, which is defined as no abnormality; both are confident diagnoses of non-malignancy. Concerns about using such non ordinal scores in ROC AUC analyses have been raised [25].

Furthermore, confidence scores for classification of abnormality in radiological studies are not based on a single characteristic, but are a composite assessment often including assessments of size, shape, morphology and other visual information [25]. The component parts of this composite score are not constrained to ensure they are used equivalently by different readers in a multi-reader study. Indeed a lack of constraint is also likely to mean that scores are used differently even within a single reader comparing two tests in the same patient. Harrington further points out that there is no evidence that confidence levels are reported in a consistent, reliable basis throughout a single experiment by all radiologists or even within each radiologist, an assumption important in ROC construction and interpretation of ROC AUC He outlines how the basis of confidence ratings vary across radiologists and lists 10 different interpretations when radiologists were asked to define “high confidence”, varying from “the image quality is good”, “the finding is obvious”, “the finding is familiar”, to confidence in their own judgement [25]. The lack of a defined objective measure of confidence means there is no standard to ensure consistency of confidence scores within or between readers across interpretations and when comparing tests. This explains why we found very different values and distributions of confidence scores in our study (Figure 2).

### Issues when confidence scores are disconnected from normal clinical practice

Confidence scores for ROC AUC analysis can be obtained either by adapting standard clinical reporting scales (e.g. BI-RADS) or by using scales specifically designed to calculate ROC AUC. The advantage of using non-standard scores is that these can be specifically designed to improve statistical power and fit with statistical assumptions. Such scales are often wide (e.g. from 1 to 100) and are clearly ordinal. However there can be problems with how such scores are used by radiologists and extrapolation to a clinical context.

When confidence scores are not based on standard clinical categories, the ROC curve does not correspond to a clinical decision curve but is based on what Dorfman et al have termed *virtual operating points* [30]. The interpretation and relevance of derived performance measures such as ROC AUC is then problematic unless the ROC curve is identical when confidence scores based on standard clinical categories are used [25]. Given the problems of interpreting ROC AUC when based on confidence scores disconnected from clinical categories, suggestions as to how to train readers to distribute their scores to reduce violation of statistical assumptions seem somewhat to have lost clinical relevance [31]. When confidence scores are disconnected from normal clinical practice there are problems in how radiologists assign such scores, particularly when the concepts underlying them may be counter to concepts used in standard clinical systems. Krupinski [33] comments on the disconnect between the confidence scores used in the “laboratory” to assess CAD assisted mammography versus the BI-RADS scales and binary endpoints used by radiologists in clinical practice. Gur [29] is similarly concerned that where there are well defined abnormalities, an attempt to use continuous confidence scores imposes a mismatch between the study and the readers' normal clinical situation. When forced to score confidence for a well defined abnormality, readers may resort to scoring on their assessment of lesion subtlety or lesion size rather than their confidence of seeing the abnormality. In support of that idea, we found an association between confidence scores and polyp size.

### ROC curves

In a study like ours, the shape of the ROC curve, and hence the value of ROC AUC is likely to be highly dependent on the scores for a few false positive results (Table 2 & Figure 5). Gur et al found curve fitting was highly dependent on the last experimentally ascertained data point [34] particularly where there are well defined abnormalities [29]. The last experimentally ascertained data points are those with the lowest specificity values and are highly influenced by confidence scores assigned to false positive diagnoses.

Different curve fitting methods can produce different curves for the same data (Figure 4), with very different resulting values for the ROC AUC (Table 2). This phenomenon can be particularly pronounced when data points are restricted to a very small part of the graph so that extensive extrapolation is required to draw the curve and calculate ROC AUC. Other researchers have also found that different methods can calculate different ROC AUC values [35] and this is recognised in FDA guidelines where it is specified that both parametric and non parametric methods should be used [6].

Some curve fitting methods failed with our data because some readers did not identify any false positive results (also known as “degenerate” data). A similar study to ours, using CT colonography to detect polyps, also experienced difficulties with fitting curves using the parametric methods included in DBM MRMC software, and reported that empirical ROC analysis was used instead [20]. However this may merely move the issues elsewhere; Gur et al found empirical non-parametric methods of fitting ROC curves highly dependent on the last experimentally ascertained data point, to a greater degree than ROC curves fitted using parametric methods [34]. Furthermore with data similar to our study, the ROC curve itself does not represent a good summary of all patient data due to the large proportion of patients with extreme confidence score values.

One way to reduce the influence of extrapolation from the study data and therefore weight the ROC AUC to the most clinically relevant data, is to use a partial ROC AUC, for example restricted to values between 75% to 100% specificity (i.e. 1-specificity in the range of 0% to 25%). However using a partial ROC AUC introduces new problems including the arbitrary choice of thresholds, which can affect which readers would have the highest ROC AUC [1]. The choice of threshold is particularly challenging to justify in a study such as ours with ten different readers and two tests, all with data points extending across slightly different ranges of specificity [34].

### Interpreting ROC AUC

Three common interpretations of ROC AUC have been proposed [36]; the average test sensitivity across all possible values of specificity; the average specificity across all possible values of sensitivity; the probability that randomly selected pairs of patients, one with and one without disease, would be ordered correctly for probability of disease.

Figure 4 illustrates why in our study we are not interested in the average value of sensitivity across all possible values of specificity, as most values of specificity do not occur in clinical practice with our test. The dots show real data corresponding to the range of thresholds at which our tests perform in clinical practice. Similarly not all values of sensitivity are used in practice. The probability that randomly selected pairs of patients, one with and one without disease, would be ordered correctly for probability of disease is not useful in clinical practice as patients do not attend outpatient clinics in pairs! [37]

ROC AUC is widely presented as a measure that avoids mistakes arising from comparing tests at single thresholds, particularly if ROC curves cross [38]. However some authors caution that when ROC curves cross or have different shapes, comparisons of ROC AUC are invalid [39]. We presented further problems with ROC AUC and its interpretation for patients in a previous article [1].

Some proponents of ROC AUC view the shift in sensitivity and specificity seen in our study as not indicating a change in diagnostic performance, because ROC curves with and without CAD overlap, and dismiss the increased sensitivity as simply corresponding to a change in test threshold. Using a microscope to identify cytological abnormalities magnifies cells and hence changes the test threshold at which abnormalities can be detected, but this threshold change is not seen as irrelevant to clinical practice. In the same way, we maintain that to change the average operating threshold of a test across readers using CAD CT colonography should not be disregarded as an irrelevance, but assessed according to its impact on the accurate diagnosis of patients.

## Conclusions

In our study comparisons of diagnostic performance derived from differences in ROC AUC led to very different conclusions than differences in sensitivity and specificity. Assigning confidence scores was found to be problematic in this detection study and the distribution of these scores was highly non-normal with the result that the ROC curve only summarised data from between 15% to 47% of the 107 patients per reader. Differences in curve fitting and methods for calculating ROC AUC led to differences in calculated values which were greater than the typical difference in ROC AUC observed in published studies. We summarise problems reported by other researchers and caution trialists to examine their study design and data to establish whether ROC AUC assumptions are likely to be met, particularly for detection studies. In our study sensitivity and specificity give a more clinically relevant summary of diagnostic performance since they are based on the diagnostic decisions used to guide clinical management.
